# Mucosal delivery of tuberculosis vaccines: a review of current approaches and challenges

**DOI:** 10.1080/14760584.2019.1692657

**Published:** 2019-12-26

**Authors:** Elena Stylianou, Matthew J. Paul, Rajko Reljic, Helen McShane

**Affiliations:** a The Jenner Institute, Nuffield Department of Medicine, University of Oxford, Oxford, UK; b Institute for Infection and Immunity, St George’s University of London, Tooting, London, UK

**Keywords:** Tuberculosis, vaccination, mucosal, aerosol, delivery, challenges

## Abstract

**Introduction**: Tuberculosis (TB) remains a major health threat and it is now clear that the current vaccine, BCG, is unable to arrest the global TB epidemic. A new vaccine is needed to either replace or boost BCG so that a better level of protection could be achieved. The route of entry of *Mycobacterium tuberculosis*, the causative organism, is via inhalation making TB primarily a respiratory disease. There is therefore good reason to hypothesize that a mucosally delivered vaccine against TB could be more effective than one delivered via the systemic route.

**Areas covered**: This review summarizes the progress that has been made in the area of TB mucosal vaccines in the last few years. It highlights some of the strengths and shortcomings of the published evidence and aims to discuss immunological and practical considerations in the development of mucosal vaccines.

**Expert opinion**: There is a growing body of evidence that the mucosal approach to vaccination against TB is feasible and should be pursued. However, further key studies are necessary to both improve our understanding of the protective immune mechanisms operating in the mucosa and the technical aspects of aerosolized delivery, before such a vaccine could become a feasible, deployable strategy.

## 1. Introduction

Tuberculosis (TB) is the most common cause of death due to a single infectious agent. There were an estimated 1.6 million deaths due to TB in 2017, and 10 million new cases [1]. TB is caused by *Mycobacterium tuberculosis* (*M.tb*) and is transmitted when a person with active disease coughs, sneezes or exhales releasing thousands of bacilli in the air. TB is primarily a disease of the lung but the bacterium can also infect other parts of the body resulting in extrapulmonary TB.

The increasing number of drug-resistant strains of *M.tb* further highlights the urgent need for an effective vaccine that could either prevent *M.tb* infection and/or TB disease. The only available vaccine against TB, Bacillus-Calmette Guerin (BCG), was developed in 1921 after the attenuation of a strain of *Mycobacterium bovis*. Although BCG is protective against childhood forms of TB such as TB meningitis and miliary disease, its efficacy is variable against adult pulmonary disease, the form responsible for the majority of incident cases [2,3]. BCG is typically administered via the intradermal route, resulting in the induction of strong systemic, but weak mucosal immune responses [4,5]. Mimicking the route of infection with vaccination, either with BCG or with a novel mucosal vaccine, might be a more successful vaccination strategy, as it would target the induction of immune responses at the point of entry of the bacteria. Furthermore, a needle-free immunization approach is safer compared to injectable vaccination. This review summarizes important immunological and practical considerations in the development of mucosal vaccines, and highlights key information gained from studies using animal experimental models.

### 1.1. Mucosa

The respiratory system in mammals can be divided into upper and lower parts. The upper parts include the nasal and oral cavities and the lower include the trachea and lung. The luminal side of the respiratory tract contains an epithelial layer of cells with tight and adherent junctions designed to control the communication between the lumen and lamina propria throughout the mucosa. The release of mucus and antimicrobial agents along with the ciliary action of epithelial cells is designed to trap, kill and remove invading pathogens [6].

The mucosal immune system can be divided into inductive and effector sites. The inductive sites are referred to as mucosa-associated lymphoid tissue (MALT), where the induction and activation of immune responses takes place. Activated immune cells then travel to effector sites via the lymphatic system in what is known as the common mucosal immune system [7,8].

MALT is composed of epithelial cells and differentiated microfold (M) cells, which sample foreign particles in the lumen before transporting to DCs on the basal side [9]. DCs will take up and process antigens before migrating back to MALT or draining lymph nodes for the initiation of pathogen-specific immune responses by T and B cells [10]. Differences in MALT between different animal species and humans exist. In rodents and non-human primates (NHP), NALT (nasopharynx-associated lymphoid tissue) is composed of paired lymphoid structures at the entrance and nasopharynx whereas in humans a more complex ring of lymphoid tissues forming a ring-like structure referred to as Waldeyer’s ring, including tonsils, allows sampling of food, air and water [11,12]. Both NALT and Waldeyer’s ring play an important part in inducing mucosal immune responses at the upper respiratory system. Bronchus-associated lymphoid tissue (BALT) however is not found in the lower respiratory tract of all mammalian species, including humans and mice, but seems to be induced by microbial exposure or other inflammatory stimulants [13]. For this reason, it is usually referred to as inducible BALT (iBALT). Apart from the conventional induction of responses in the lymph nodes, the contribution of iBALT in generating antigen-specific immune responses, particularly during influenza infection, is now appreciated [12]. In the absence of lymph nodes, iBALT formation was enough to control *M.tb* infection in the lungs of mice [14]. Large areas of organized iBALT that surround granulomas are present in monkeys with latent *M.tb* infection but not with active disease and also in humans [15]. In an NHP model of TB/simian immunodeficiency virus (SIV) co-infection, a number of animals were able to prevent TB reactivation despite the lack of CD4+ cells [16]. The presence of memory CD8 + T cells and iBALT correlated with this observation. Protection induced by aerosol vaccination with an attenuated *M.tb* strain was also associated with the generation of iBALT in NHP [17].

In addition to differences in the structure of the MALT, evolutionary pressure exerted by rapid pathogen adaptation as well as functional redundancy in immune effectors has led to significant biases in the immune response profile between mammalian species. These differences complicate model selection and necessitate routine testing of candidate mucosal vaccines in multiple models to support progression to clinical trials. Comparison between bronchoalveolar lavage (BAL) cells in cynomolgous macaques and humans indicated a large degree of similarity between the two species [18]. This, in combination with the ability of these animals to develop both active and latent infection after low-dose *M.tb* exposure, makes them a good model to study vaccine immunogenicity and efficacy [19]. However, 50% of the infected animals will go on to develop active disease, a situation different to humans. In addition, the high cost of NHP experiments justifies the testing of only the most promising vaccine candidates [20]. As a result, smaller animal models are still necessary for early-stage vaccine efficacy testing.

#### 1.1.1. Innate immune responses

The nonspecific and specific beneficial effects of BCG have highlighted the important role of trained innate immunity in the control of infections [21,22]. More recently group 3-innate lymphocyte cells (ILC3) have been shown to play an important role in controlling *M.tb* infection in mice [23]. Whether and how these cells, and other ‘trained innate immunity members’ can be exploited by vaccination remains to be determined. Donor-unrestricted T cells such as mucosally associated invariant T cells (MAIT), γδ T, and invariant natural killer cells (iNKT) have also been shown to have important roles in immunity to *M.tb* infection [24–27]. In particular, MAIT cells, which are situated mostly at mucosal sites, are activated by both *M.tb* infection and BCG vaccination in NHPs [28]. If they are confirmed to be protective, mucosal vaccines targeting MAIT cells, e.g. using MAIT antigens, could potentially improve early control of infection.

#### 1.1.2. Adaptive immune responses

Humoral immune responses in the mucosa are mainly mediated by secretory IgA (sIgA), which is considered the hallmark antibody. sIgA is resistant to proteases and functions by neutralizing pathogens, toxins, and allergens and it also has anti-inflammatory functions [29,30]. Systemically delivered vaccines are generally poor inducers of sIgA at mucosal sites, as upregulation of mucosal homing receptors on cognate B cells is efficient only when priming occurs in MALT inductive sites [29]. Although the role of antibodies in TB protection remains unclear, there are a number of studies to suggest an important role for sIgA. For example, mice deficient in either IgA or sIgA through deletion of the polymeric IgA receptor were less able to control mycobacteria compared to wild-type controls [31,32]. The most likely mechanism of action seems to be the opsonization of bacilli and therefore antibodies that recognize antigens on the bacterial surface might be more effective in controlling disease [33–35]. In human FcalphaR (CD89) transgenic mice, intranasal instillation of an immunotherapy containing human IgA recognizing the exposed antigen Acr (hspX) and IFN-gamma, reduced bacterial burden when treatment was given either before, during or up to a week post challenge (manuscript in preparation, Dr Rajko Reljic, St George’s University of London, personal communication). This protective effect was previously shown to depend on both components of the immunotherapy and the use of the CD89 transgenic mouse line [36]. The capacity to induce mucosal IgA to surface antigens is therefore likely to be a feature of an effective mucosal vaccine.

A recently discovered population of non-circulating resident memory B cells in the lungs (Brm) have been shown to play an important protective role during influenza infection in mice [37]. The generation of and importance of this cell population in *M.tb* infection remains to be determined; however, it is important to note that antigen-encounter was required for Brm lung localization. This might mean that respiratory rather than systemically administered vaccines will be more likely to generate this cell population.

**Figure 1.**
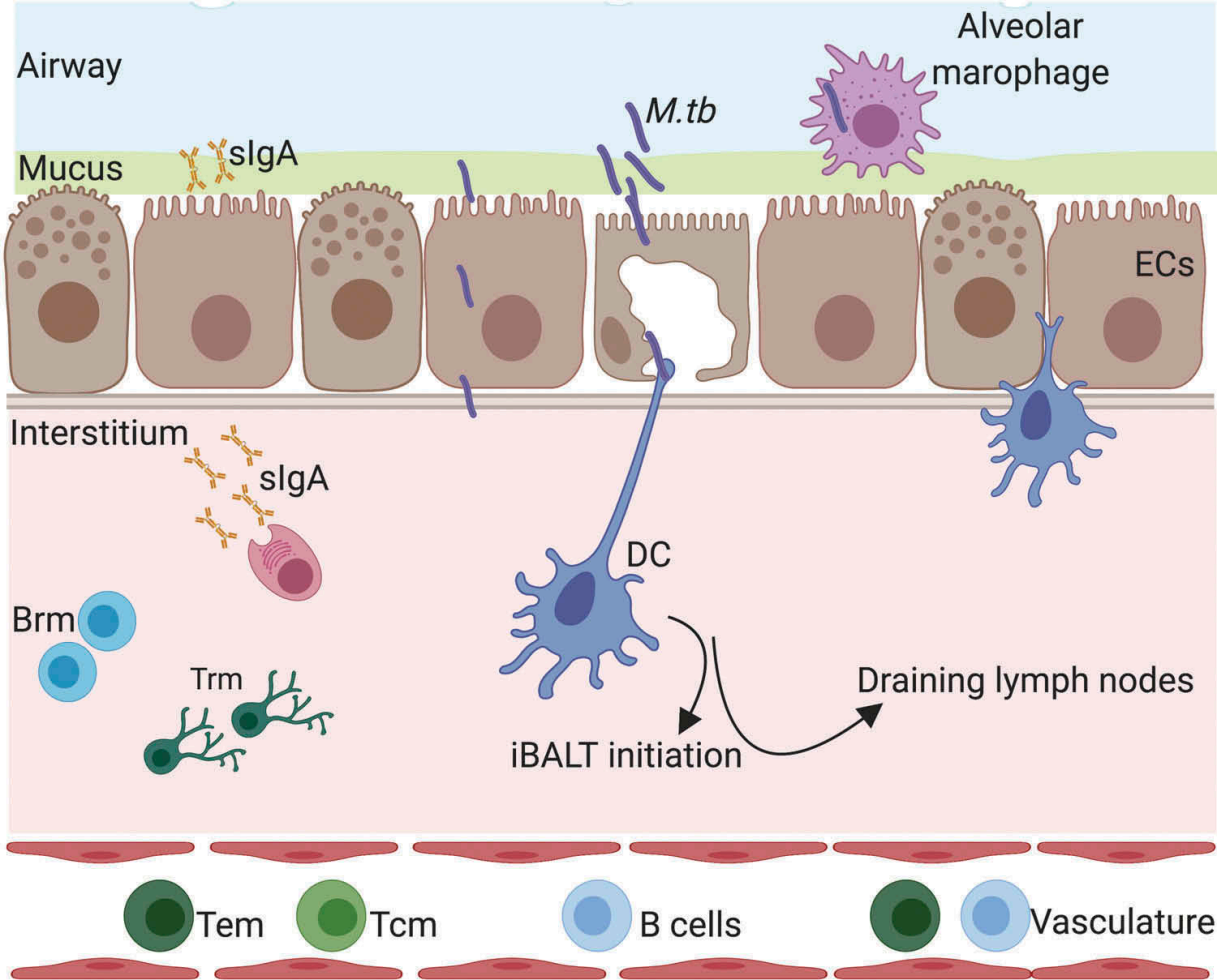
Respiratory mucosal responses following *M.tb* infection or mucosal vaccination. DC = dendritic cells, EC = epithelial cells, sIgA = secreroty IgA, Brm = resident memory B cells, Trm = resident memory T cells, *M.tb*= Mycobacterium tuberculosis, iBALT = inducible bronchus-associated lymphoid tissue. Created with Biorender.com.

Cellular immunity is of paramount importance for TB control but these responses do not always correlate with protection [38–43]. Vaccination induces memory immune cells that can quickly differentiate and expand upon infection [44]. It is generally accepted that there are two populations of memory cells, effector (Tem) and central (Tcm) [45]. These populations are characterized by differential expression of lymphocyte-endothelial cell adhesion molecule CD62L and the chemokine receptor CCR7, with Tcm (CD62L^lo,^ CCR7^hi^) cells restricted to circulation through the lymphatics and vasculature, while the Tem (CD62L^hi,^ CCR7^lo^) population interacts with non-lymphoid tissue. Mucosal immunization approaches are efficient at inducing significant Tem and Tcm at primary lymphoid sites [17,46]. However, more recently a memory population that permanently resides in non-lymphoid tissues has been identified, termed as resident memory cells (Trm) [47,48]. With the help of the intravascular (iv) staining technique, that discriminates cells that are in the interstitium (iv-) from cells in circulation (iv+), Trm have also been identified in the lungs [49–52]. They have subsequently been shown to play an important role in protecting against influenza infection in mice [50]. The site of infection seems to define the location of these cells, highlighting that matching the route of vaccination to the route of infection might be optimal for their generation [53]. In *M.tb* infection, iv- CD4+ cells isolated from the lung were more protective compared to the corresponding iv+ population [54]. There are some reports to show that the life span of these cells is shorter compared to Trm in other locations such as the skin [55]. In a study by Bull et al., the intranasal administration of BCG was more protective compared to the systemic route [5]. This superior protection was associated with a higher number of Trm, with both declining at a later time point post vaccination, in agreement with the influenza data. Approaches designed to supplement vaccine profiles with lung Trm using subunit vaccines have been met with mixed results. Using the H56:CAF01 in a systemic prime-mucosal boost schedule resulted in a significant enhancement of long-lived lung Trm cells and the promotion of early T cell responses in the lungs of challenged mice [56]. However, the presence of ESAT-6 specific lung CD4+ Trm was not associated with improved bacterial control, and furthermore, control of infection was found to be sensitive to blocking recruitment of leukocytes from the systemic compartment with FTY720. In contrast, pulmonary immunization with recombinant influenza virus expressing *M.tb* peptides induced TB-specific CD4^+^ Trm and protection that was insensitive to extensive treatment with FTY720 [57]. The presence of vaccine-elicited CD8+ lung Trm has been associated with protection in other studies [46,58]. Further work with other vaccine candidates is therefore needed to establish the circumstances in which Trm may contribute to protection and establish other important characteristics of the Trm response, such as their life span. A summary of some of these responses induced by *M.tb* infection is shown in Figure 1.

Mucosal vaccination has been shown to induce higher levels of IL-17 compared to the systemic route and to be important for protection. Mucosal administration of BCG in mice and NHP resulted in IL-17 responses that were associated with protection [59–62]. In addition, mucosal adjuvants such as cholera toxin, monophosphoryl lipid A (MPL) with chitosan and type II heat liable enterotoxin (LT-IIb) have been shown to induce potent IL-17 responses and improve protection [62–64].

## 2. Mucosal vaccines

### 2.1. Mucosal vaccines for other diseases

A number of successful mucosal vaccines against other diseases are already in widespread use, emphasizing the feasibly and safety of this route. These include vaccines against polio, cholera, rotavirus, salmonella, and influenza. Vaccines against the first four diseases are administered via the oral route whereas for influenza via the nasal route. Currently, all mucosal vaccines are whole cell or whole virus preparations, either attenuated or inactivated, and only one (Dukoral™ OCV) given in formulation with an adjuvant cholera toxin B subunit (CTB). Interestingly, inactivated polio vaccine (IPV) is less effective than attenuated polio vaccine (OPV) in inducing mucosal immunity in the gut [65], suggesting that the interaction of the attenuated virus with the host contributes to building an effective immune response. The addition of CTB in the Dukoral formulation is of uncertain benefit; however, Dukoral has been reported to offer better immediate protection than non-adjuvanted OCVs [66]. The current mucosal influenza vaccines, MedImmune’s FluMist™ and FluMist Quadrivalent™, and AstraZeneca’s FluEnz Tetra™ consist of live, attenuated viruses and have clearly demonstrated efficacy against some targeted influenza strains, although not all [67]. Recombinant subunit influenza vaccines have been developed for systemic application either with (Sequirus FluAD™) or without adjuvants (Sanofi-Pasteur FluBlok™). The efficacy of unadjuvanted formulation has attributed in part to the efficient formation of virus-like particles (VLPs), which are efficient activators of APCs and promote cross-presentation [68]. The aerosol route of vaccination is also been investigated for measles. Although the systemic route of the measles vaccine is safe and effective, it is believed that aerosol administration might increase vaccine coverage due to its ease of administration and reduced risks associated due to the lack of needles. Results from a number of studies demonstrated the safety of this approach and identified the aerosol route to be at least as efficacious as the subcutaneous route [69]. The immunogenicity of the aerosol route, based on seropositivity and seroconversion, was shown to be inferior in infants 9–12 months old, compared to the subcutaneous route [70]. Whether this observation can be attributed to the route or administration, e.g. duration of nebulization or breathing rate is yet to be determined. However, the aerosol route could be used to more efficiently boost previous immunity induced by subcutaneous measles vaccination, as previously shown in schoolchildren [71]. Lessons learnt from the development of these mucosal vaccines can inform the design of TB vaccines targeting the respiratory mucosa.

Aerosolized vaccine delivery could be potentially also applicable to infants from the age of one year and above, as demonstrated by the aerosolized measles (1 y) and flu (2 y) vaccines. Younger infants, on the other hand, might require substantial further technological adjustments in order to dose the vaccine correctly and this approach may not be as suitable. However, considering that BCG vaccine will continue to be recommended to neonates for the foreseeable future, the aerosolized vaccine approach would most likely be best suited for a booster vaccination later in infancy/childhood or in adolescence/adulthood.

### 2.2. Mucosal vaccines for TB

There are currently no confirmed immune responses that correlate with protection against TB. This represents a big obstacle in the vaccine development field for all types of TB vaccines and routes. In the absence of a surrogate marker of protection efficacy, experiments in animal models are a necessary requirement.

#### 2.2.1. BCG and attenuated whole-cell live vaccines

There is a body of evidence supporting the safety of orally delivered *M. bovis* BCG substrain Moreau Rio de Janeiro due to its long-term historical use in Brazil, and more recent clinical studies in the UK [72,73]. A single dose of oral BCG was found to boost PPD-specific IFNγ responses in previously BCG i.d. vaccinated individuals for at least 3 months after vaccination. It is interesting to note that this particular substrain of BCG carries a unique RD16 region, which has been associated with its safety profile when delivered orally [72]. The oral *vs* intradermal administration of BCG Danish was also investigated in a small-scale trial in humans with no detectable adverse effects [4]. Although intradermal BCG induced stronger immune responses in the blood, oral BCG resulted in stronger mucosal responses in BAL and secretory IgA in nasal washes and tears. Oral vaccine administration is an attractive route mainly due to the ease of administration resulting in improved compliance but also due to the ability to induce responses at distal mucosal sites [4]. However, developing an oral vaccine has its challenges as it needs to be robust enough to withstand the harsh acidic environment in the stomach and requires the inclusion of an adjuvant to reduce the risk of tolerance [74]. Since *M.tb* primarily infects the lung, matching the route of infection to the route of vaccination might be a more effective approach when developing TB vaccines. The oral route might be more efficient as a boost to a prior respiratory or systemic vaccination [75].

Studies investigating the aerosol delivery of BCG using guinea pigs date back to the 1950s [76]. Since then there has been accumulating evidence in mice to further highlight the superiority of the respiratory route of BCG immunization in controlling *M.tb* infection [5,58–60,77,78]. This finding was confirmed both in guinea pigs and NHP [79–81]. NHP vaccinated with intratracheal (i.t.) BCG as a boost to a previous intradermal (i.d.) BCG vaccination had a reduced level of pulmonary disease compared to i.d. BCG after aerosol *M.tb* infection [82]. Endobronchial instillation of BCG was able to protect rhesus macaques, significantly more than systemic administration [83]. More recently, BCG administered endobronchially protected NHP from a repeated low-dose *M.tb* infection [60]. NHP that were vaccinated mucosally had a higher proportion of CD4+ IFNγ^+^/TNFα^+^/IL2^+^/IL17^+,^ CD8+ IFNγ^+^/TNFα^+^ and more T cells in their bronchoalveolar lavage (BAL). In addition, granzyme B, IL10, granulocyte-macrophage colony-stimulating factor (GM-CSF) and levels of IgA were higher in the mucosal compartment after mucosal immunization compared to the intradermally immunized group. Polyfunctional Th17 cells, IL10 and IgA correlated with the protective efficacy observed in this study.

In addition, in cattle, the simultaneous administration of BCG endobronchially and systemically, resulted in significant protection against *M.bovis* infection compared to unvaccinated control animals [84].

In humans, aerosolized BCG was first administered to children and young adults without reported side-effects in the 1960s [85]. Ongoing clinical trials are currently evaluating aerosol BCG administration, in healthy BCG-naïve adults (NCT02709278 NCT03912207). To date, this route of immunization is well tolerated with no Serious Adverse Events (Prof. Helen McShane, University of Oxford, personal communication).

To date, BCG remains the only whole-cell vaccine to be investigated as a mucosal vaccine. Genetically engineered live vaccines have been demonstrated to outperform BCG in preclinical studies when delivered systemically [86,87]. Development of a rationally designed whole-cell vaccine candidate specifically for the mucosal route is an intriguing possibility, and approaches could include engineering of overexpressed mucoadherins alongside modifications made to optimize antigen presentation. For example, exposure of BCG to human alveolar lining fluid, to expose underlying epitopes, resulted in improved BCG protection in mice [88]. Selective removal of inflammatory lipids from BCG rendered it more protective and resulted in less lung pathology compared to conventional BCG when delivered mucosally in mice [89].

Due to the protective effects against childhood TB manifestations, the systemic route of BCG administration is unlikely to be abandoned in the future. Even so, studies described above highlight the advantages of pursuing novel live vaccines for delivery via the mucosal route.

#### 2.2.2. Viral vectors

Although the efficacy result from the first prophylactic vaccine in clinical trials, MVA85A, was disappointing, this vaccine has an excellent safety record [90–93]. The safety of MVA85A allowed its aerosol administration in humans, the first TB vaccine to be evaluated using this route of administration in humans [94]. MVA85A was administered in BCG-vaccinated healthy volunteers either via aerosol or intradermally. Volunteers that received the aerosol vaccination had stronger mucosal and equally strong systemic responses compared to the intradermal group. Interestingly the mucosal route had a lower level of systemic humoral anti-vector responses. In a follow up phase I experimental medicine study, it was hypothesized that the lower levels of anti-vector responses would result in improved Ag85A-specific responses in a sequential homologous prime-boost immunization [95]. Surprisingly there was no boosting of antigen-specific responses in the aerosol-intradermal group, in contrast to the intradermal-aerosol group were a boosting effect was detected. However, whereas aerosol followed by intradermal MVA85A was well tolerated, the intradermal-aerosol group was associated with transient but significant respiratory side effects. The reasons behind these observations are not known but these data are important to the development of aerosolized vaccines. It is worth noting that preliminary data from the aerosol administration of MVA85A to adults with latent TB were not associated with any safety concerns (ClinicalTrials.gov NCT02532036). In mice, intranasal administration of MVA was able to induce iBALT in the lungs [96].

Adenoviral vectors have been widely used for intranasal administration mainly due to their natural tropism for the respiratory epithelium. These viruses are typically rendered replication-deficient and modified to express different mycobacterial antigens [97]. Advantages include their large antigen packing capacity and their intrinsic adjuvanticity [98]. In addition, they have an excellent safety record in humans [99,100]. Examples include adenovirus type 5, expressing Ag85A (AdHu5.85), and adenovirus type 35 that have both completed phase I clinical trials [100,101]. In cattle, the simultaneous delivery of systemic BCG with endobronchial AdHu5.85A resulted in a lower number of animals with visible pathology and granulomata, compared to naïve control animals [84]. Interestingly, in mice, the intranasal route of AdHu5.85 was more protective compared to the intramuscular route however differences between the two routes were not as striking in NHP [102]. Recently, an AdHu5-based vaccine candidate failed to enhance BCG mediated protection in Rhesus macaques when delivered simultaneously by the aerosol and intramuscular routes, despite eliciting robust lung T cell responses [103]. Due to the presence of neutralizing antibodies against adenoviruses, chimpanzee adenoviruses are now utilized as safe and effective alternative approach [104,105]. The intranasal delivery of chimpanzee adenovirus expressing Ag85A (ChAdOx1.85A) boosted with modified vaccinia Ankara 85A, improved the efficacy of BCG-primed mice [106]. Aerosolized ChAdOx1.85A is now in phase I clinical trials [107] (Prof. Helen McShane, University of Oxford, personal communication). Other i.n. chimpanzee adenoviruses that have shown promise in mice include ChAdOx1.PPE15 and AdCh68.85A [108,109]. Other viral-based vaccines that have shown promise include recombinant influenza virus expressing mycobacterial antigens [57].

#### 2.2.3. Protein-adjuvant vaccines

Non-viral vaccines are more challenging to develop for mucosal administration. In part, this is due to the need to formulate subunit vaccines to withstand the physical and chemical barriers presented at the mucosa but also due to a dearth of suitable adjuvants. There are currently no adjuvants with regulatory precedent for intranasal/aerosol delivery in humans. However, some compounds have entered clinical studies designed to investigate their potential as mucosal adjuvants (Table 1). Many others are currently under testing in animal models with some promise [110]. Adjuvants such as Bis-(3′,5′)-cyclic dimeric guanosine monophosphate (c-di-GMP) and flagellin have been shown to induce strong mucosal immune responses [111,112].

**Table 1.** Mucosal adjuvants in human clinical trials. Vaccine clinical trials registered on clinicaltrials.gov were searched for compounds categorized as adjuvants and delivered to mucosal surfaces.

Compound	Antigen	Phase	Results	Investigators	Ref	Trial Id
*Type 1 IFN*	Influenza, 2006–2007 trivalent inactivated vaccine	1	No effect	NIAID, Baylor College of Medicine	[113]	NCT00436046
*E.coli LT*	Influenza, virosome/subunit	n/a vaccine used in Switzerland in 2000–2001	Effective, but strong association with Bell’s Palsy	Berna Biotech	[114]	
*polyICLC (Hiltonol)*	Respiratory viral infections, including influeza	1		NIAID		NCT00646152
*polyI:polyC12U (Ampligen)*	Influenza, FluMist	I/II Canceled due to withdrawal of FluMist in 2016	Specific SIgA titers >4x over baseline. Well tolerated	Hemispherx Biopharma Inc.	[115]	NCT01591473
*Oleic acid, mono-olein* *(Endocine™)*	Influenza A/California/07/2009 H1N1	I		Eurocine Vaccines Ab		NCT02998996
*Oleic acid, mono-olein* *(Endocine™)*	HIV Vacc-4x p24 consensus peptides	I	Safe, antigenic dose-dependent immune responses	Eurocine Vaccines Ab	[116]	NCT01473810
*Cholera toxin B subunit*	None					NCT00820144
*Aluminum* *hydroxide*	Streptococcus pneumoniae GEN-004	I	Non-significant reduction in carriage rate	Genocea Biosciences		NCT02116998
*Rintatolimod*	FluMist	I/II	Well tolerated. 92% of vaccinees produced specific sIgA.	Hemispherx Biopharma Inc.		NCT01591473 (terminated as nasal flu spray not CDC recommended 2016–2017)
*Proteasome (*N. meningitides *OMP*)	Trivalent Influenza HA			ID Biomedical, Quebec	[117]	NCT02522754^1^

Other approaches for effective vaccination in the airways include active targeting of vaccines and/or formulation of particulates for more efficient uptake by antigen-presenting cells. Examples include liposomes, virus-like particles (VLP) and nanoparticles. A DNA vaccine encoding heat shock protein (hsp65) encapsulated in cationic liposomes was more efficient when administered i.n. compared to the i.m. route [118]. Intranasal delivery of wax nano-particles conjugated to Ag85A-HBHA, for specific targeting to heparin on epithelial cell was safe and protective in mice [119]. Spores from *Bacillus subtilis* have been used as an adjuvant and antigen-coating vehicle for the delivery of a fusion protein FP1 (Ag85A-Acr-HBHA) to the lungs of mice [46]. This candidate vaccine significantly reduced post-challenge bacterial burden in the lungs of BCG-vaccinated and unvaccinated guinea pigs and mice when delivered with the TLR3 ligand polyI:C [46] (Dr. Simon Clark, PHE, Prof. Martin Rottenberg, Karolinska Institute, personal communication), and induced a significant expansion of Trm cells in the lung. This candidate is currently under evaluation in the NHP challenge model (Dr. Rajko Reljic, St George’s University of London, personal communication). Data from the NHP model for mucosal subunit vaccines are currently extremely limited. In cynomolgus macaques, aerosol delivery of H56 (Ag85B, ESAT6 and Rv2660) in formulation with CAF01 liposomes resulted in increased bacterial clearance compared to intranasal or intramuscular administration, although other aspects of disease pathology did not differ between the vaccination routes [120]. Table 2 lists mucosal prophylactic TB vaccines that have been tested in multiple animal models or human clinical trial(s), along with some selected other studies.

**Table 2.** Experimental mucosal prophylactic vaccines for TB. Vaccines tested in multiple animal models or human clinical trial(s) are listed along with selected other studies.

Vaccine	Mouse	Guinea Pig	Cow	NHP	Human Clinical Trials
**Viral Vectored Vaccines**					
*MVA85A*(University of Oxford)	**Intranasal** boost enhances BCG protection [121]			**Aerosol** boost immunogenic [141]	**NCT01954563** [95] **NCT01497769** [94] **NCT02532036** – terminated poor recruitment
*Ad5Ag85A*(McMaster University)	**Intranasal** boost enhances BCG protection [122]	**Intranasal** boost to BCG improved survival of vaccinated animals compared to control BCG [123]	**Endobronchial** instillation. Similar immunogenicity to intradermal [124]		**NCT02337270** – recruiting since Jan 2015
*ChAOx1.85A*(University of Oxford)	**Intranasal** boost with MVA85A enhances BCG protection [106]				**Aerosol** Phase I clinical trial in Lausanne (BASEC ID: 2017–02308) ^97^
*ChAdOx1.PPE15* (University of Oxford)	**Intranasal** boost enhances BCG protection [109]	**Intranasal** BCG boost improves CFU counts in spleen (S.Clark, *pers. Comm*, manuscript in preparation)			
*AdCh68.85A*(McMaster University)	**Intranasa**l boost enhances BCG protection [108]				
**Subunit vaccines**					
*Nano-FP1 (Ag85B-Acr-ESAT6-HbHA adsorbed carnauba wax nanoparticles): polyIC*	**Intranasal** boost enhances BCG protection [131]	**Endobronchial** BCG boost reduces disease (M. Rottenberg *pers. Comm*)			
*Spore-FP1 (Ag85B-Acr-ESAT6-HbHA adsorbed onto Bacillus subtilis spores): polyIC*	**Intranasal** Bacterial burden in lungs reduced after homologous i.n. vaccination or BCG boost [46]	**Endobronchial** vaccination reduces disease (S. Clark *pers. Comm*. Manuscript in preparation)		**Aerosol** boost immunogenic (A. White *pers. Comm*)	
*H56:CAF01*	**Intranasal** boosting immunogenic but protection not superior to systemic [56]				
**Whole cell vaccines**					
*BCG*	**Intranasal BCG** [125] **Intranasal BCG + CT** [62]	**Aerogenic** [79]	**Aerosol** [126]	**Aerosol [127]** (immunogenicity only)	**NCT02709278** – status unclear **NCT03912207** – recruiting April 2019
*MtbΔSigH*	**Intranasal** Establishes infection with reduced lethality to wild-type [128]			**Aerosol** vaccination protective (superior to BCG) [17]	
*TBvac85* *(M. shottsii/Mtb Ag85B)*		**Intranasal** protection equivalent to intradermal BCG [129]			
*Salmonella/Ag85B-ESAT6*	**Oral** prime immunogenic [130]	**Oral** prime systemic boost regime equivalent to BCG [130]			

## 3. Challenges

Targeting the respiratory mucosa is a relatively new field in human vaccination and there are therefore a lot of uncertainties when choosing the route and method of administration, precise dosing, and vaccine composition.

### 3.1. Aerosolization

An effective method for the aerosolization of a vaccine candidate needs to be reproducible both in its effect on the vaccine itself and the quality of the aerosol it generates. Various types of equipment exist for the nebulization of liquid for inhalation without heating, based on air pressure, ultrasonics or vibrating meshes. Shear forces or heat generation by any of these techniques can rapidly degrade proteinaceous material, and the resulting aggregation then rapidly decreases device performance and aerosol quality. Wax nanoparticles have been shown to protect a TB antigen fusion protein during aerosolization with a vibrating mesh nebulizer (Omron U22) [131]. Aerosols can also be created from a dry powder using appropriate excipients to avoid physical damage to the vaccine [132]. The dry-powder approach has been used to vaccinate mice *via* the pulmonary route with a vaccine based on the cutinase-like protein (Culp) and a TLR2 agonist with positive results [133]. The WHO aerosol measles vaccine effort, having established an effective aerosol immunization approach based on liquid inhalers, is now looking toward dry-powder inhalers to boost vaccination rates by increasing the cost-effectiveness of the vaccine [134].

### 3.2. Physical barriers

A vaccine has to be robust enough to withstand physical barriers presented at the mucosa, such as pH differences, enzyme degradation and overcoming the mucocilliary action for deposition on the mucosal surface.

### 3.3. Deposition

**Figure 2.**
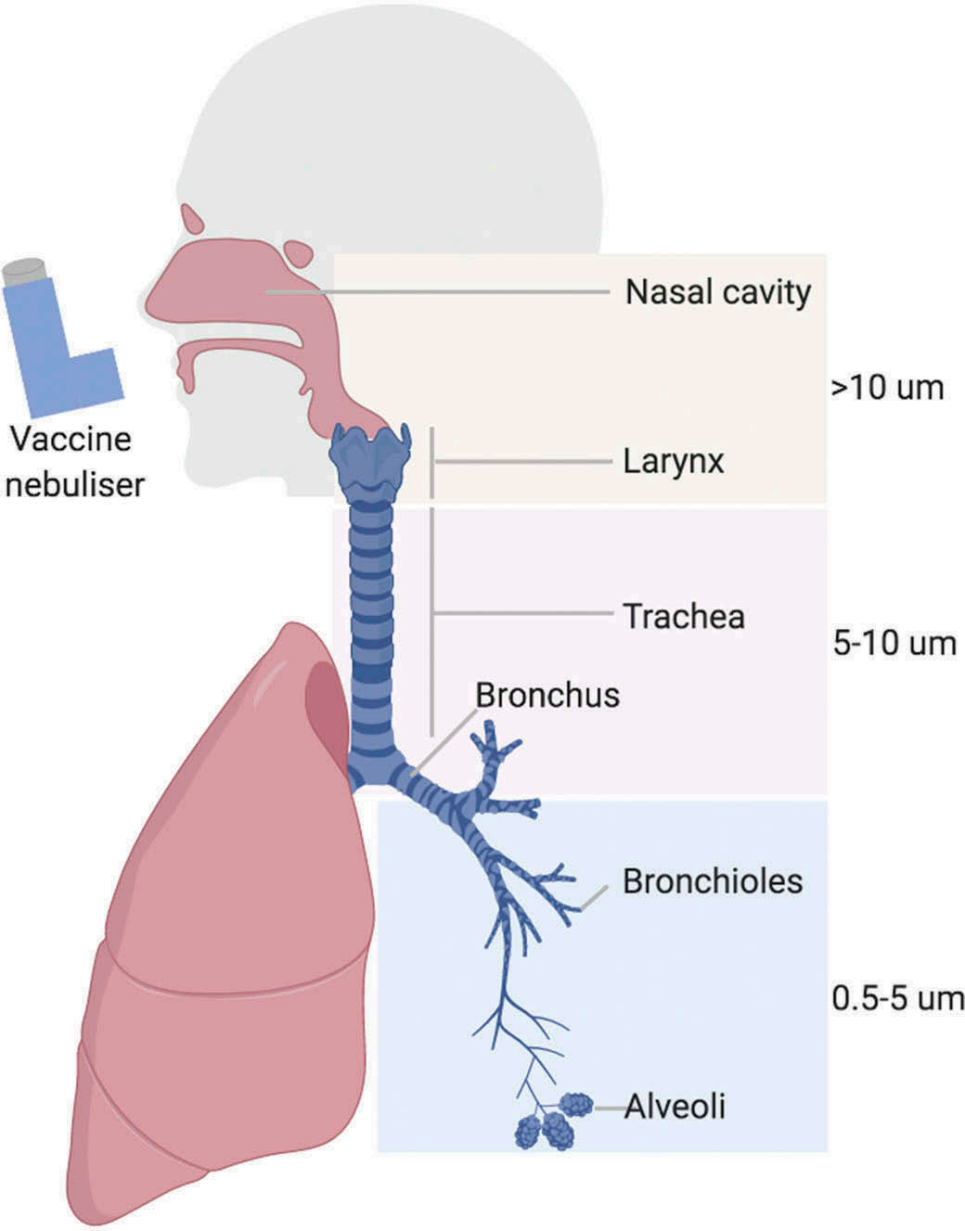
Respiratory system in man and vaccine deposition following aerosol delivery (created with BioRender.com).

The intranasal, endobronchial and intratracheal routes are normally used in pre-clinical studies but more translatable approaches such as intranasal or aerosolization would be more appropriate for humans. However, the choice of administration will have an effect on the location of the induced immune response. For example, an intranasally administered vaccine is more likely to induce immune responses locally and in the upper airways [135]. In contrast, an aerosolized vaccine might have the capacity to get deposited in different parts of the respiratory tract depending on particle size (Figure 2). Targeting different lung regions depends on particle size as well as breathing pattern [136]. As a rough guide particles of 0.5–5 μm will be deposited in the bronchotracheal and alveolar space, whereas larger sized particles up 10 μm are more likely to be deposited in the upper airways [137]. The majority of *M.tb* particles generated when an infected person coughs or sneezes are under 100 μm, with 0.5–5 μm being the most effectively transmitted [137,138]. Nevertheless, larger size particles can still result in disease and therefore a mucosal vaccine that results in the generation of broader size particles might be likely to induce responses to the sites of mycobacterial deposition. The surface tension of droplets is also a factor influencing spreading of formulation after deposition in the airways [139].

### 3.4. Dose

Variations in breathing rate, vaccine losses during administration and the inability to precisely quantify the amount of vaccine administered to the lungs are likely to result in a higher variability in the immune responses induced. Losses in aerosolization are typically high. In one study of albuterol administration, the maximum achieved proportion of inhaled and retained drug was 14%, and humidity and patient respiratory impairment was identified as factors likely to increase variability [140]. Nebulizers have been successfully used to administer viral vector vaccines and BCG, in NHP and in clinical trials, with loses ranging from 0% in viral vectors to 50% with BCG [94,141] (Stylianou E. et al., unpublished data). Studies using the mucosal route of administration should therefore make an appropriate allowance for this variability in power calculations and when interpreting data.

## 4. Microbiome

Recent evidence has suggested a possible role of the microbiome on mucosal vaccine efficacy [142,143]. It has been reported that the immunogenicity and efficacy of orally administered vaccines tend to be lower in low and middle-income countries. For example, the orally administered rotavirus vaccine efficacy ranges from 90% in high-income to 39% in low-income counties [144–147]. Higher levels of *Actinobacteria* were associated with higher responses to BCG, oral polio and tetanus toxoid vaccine, whereas *Enterobacteriales, Pseudomonadales*, and *Clostridiales* with lower responses in Bangladeshi infants [148]. The important role of the gut microbiome on distal sites such as the lung is now beginning to be better understood [149]. Helminth infections and *H.pylori* have been shown to affect both BCG-induced immune responses and have a potential effect on disease control [150–153]. In a cohort of household contacts living with an active TB patient, MAIT cell number and function correlated with the presence of specific gut microbes [25]. The relationship between the gut microbiome and MAIT cell generation was further emphasized in a mouse model of microbial dysbiosis [154]. Here, a healthy gut microbiome was associated with MAIT cell accumulation in the lungs and subsequently better early control of *M.tb* infection.

Although to date, the majority of studies have focused on the gut and the gut microbiome, next-generation sequencing revealed the presence of a microbiome throughout the respiratory tract [155]. These microbes have a huge impact in controlling respiratory infections from progressing from the upper to the lower respiratory tract. The presence of different microbes together with *M.tb* and how this interaction can influence *M.tb* control is now beginning to emerge [156]. Whether the lung microbiome has a role on mucosal vaccine efficacy and immunogenicity is yet to be determined.

## 5. Conclusion

Mucosal vaccination, a needle-free vaccination approach offers many advantages over traditional systemic routes, such as safety and ease of administration the later of which could improve compliance and result in higher vaccine coverage. The mucosal route is particularly attractive for TB vaccines due to the induction of immune responses in the lung, the primary site of *M.tb* infection. There are however also a number of challenges associated with this approach. Some of these include the need for effective adjuvants but also the difficulty in replicating the route of aerosol vaccination in humans to small animal models. Nevertheless, there have been significant steps in recent years with a number of candidate mucosal vaccines already tested in clinical trials that highlight the safety and feasibility of this route.

## 6. Expert opinion

There are undoubtedly both immunological and practical advantages in mucosal delivery of a TB vaccine. By targeting respiratory mucosa and the lungs, a mucosal vaccine is more likely to induce a protective immune response at the site of infection than a parenterally administered vaccine. By avoiding the use of needles and the attendant risks of pathogen cross-contamination, a mucosal vaccine would be easier to administer in large vaccination campaigns. Importantly, successful mucosal vaccines against other respiratory pathogens such as influenza and polio have established an important precedent, lending further support to the feasibility of a mucosal TB vaccine approach. However, this approach is not without its challenges and consequently the progress so far has been relatively slow. Some of those challenges are not unique to mucosal vaccines *per se* but apply to TB vaccines in general, namely, the lack of immune correlates of protection, inadequate experimental animal models and insufficient understanding of protective mechanisms involved. Others are specific to the mucosal approach and involve the lack of understanding of the mucosal immune mechanisms and the unresolved technical aspects of mucosal vaccine delivery. Most licensed mucosal vaccines utilize the oral route but with the exception of the orally administered BCG this route is probably not feasible for a new TB vaccine. Likewise, most animal studies of mucosal vaccine candidates involve intranasal or intratracheal instillation of the vaccine in liquid form, yet this is not a feasible route for human application. In humans, a mucosal TB vaccine is almost certainly likely to be delivered in some form of aerosol (wet or dry) through the mouth or the nose. This poses a challenge when validating mucosal vaccines in animal studies. Aerosolized vaccine delivery is not practical in small animal models (mice and guinea pigs), leaving only the costly and not easily accessible nonhuman primate (NHP) model to perform such studies. Indeed, most of the current knowledge of aerosolized TB vaccine delivery comes from a limited number of NHP studies using BCG or viral vector vaccines. What transpired from these studies is that it is very difficult to control the delivered dose in the lungs and even more difficult to control the vaccine distribution throughout the respiratory system. Even before getting into the lungs, vaccine may be negatively affected by the process of aerosolization. Furthermore, very little is known about aerosolization of protein- and other types of vaccine formulations. This means that there is a clear need to improve and understand better the technical aspects of aerosol delivery as well as the aerobiology of the lungs. We also need to improve our understanding of the immune mechanisms, both innate and adaptive, operating in the respiratory mucosa, so that they can be optimally exploited to the vaccine’s benefit. Perhaps such detailed studies of immune mechanisms should be performed only after the proof of concept has been firmly established that a mucosal vaccine is protective and that this route of immunization is superior to the systemic route. In that regard, the current evidence from small animal models and NHP is encouraging although not yet entirely compelling and consequently more studies need to be performed, especially in NHP and possibly also in humans. When it comes to NHP, the main challenges are to improve the efficacy of aerosolized delivery and also register a more profound protective effect than has been observed so far. To achieve the latter, one approach could be to circumvent the aerosolized delivery and the associated dosing issues by presenting the vaccine directly to the lung mucosa by bronchoscopic instillation, as it was done by Dijkman et al. [60]. While not a deployable route in humans, this route of delivery can help establish the proof of concept that mucosal-vaccine-induced immunity can be more protective than that induced by parenteral immunization. If so, then it would be fully warranted to pursue the aerosolized approach and try and reproduce such protection by a more deployable route such as aerosol.

In conclusion, much progress has been achieved with mucosal vaccine approaches in TB but significant challenges still remain to make this into a successful vaccine strategy. In our view, further progress in mucosal TB vaccination approach could be achieved by focusing on the following priorities, which we outlined at the recent meeting of the Aerosol and Mucosal Vaccination group at the CTVD (Collaboration for TB Vaccine Discovery) meeting in Seattle, at the Bill and Melinda Gates Foundation:

- Establishing firmly if aerosolized BCG vaccination is superior to intradermal in NHP studies
- Performing proof of concept human studies with aerosolized BCG
- Optimizing experimental models and delivery systems for different mucosal vaccine formulations (live organisms, viral vectors, and protein vaccines)
- Better understanding of the mechanisms of protective innate and adaptive immunity in the lungs
- Better understanding of the aerobiology of the lungs and respiratory vaccine delivery, in terms of aerosol particle size, distribution and viable vaccine recovery.
- Translating experimental models of mucosal delivery to potential human application and back translating human findings into preclinical studies to further improve models

## Article highlights

- Matching the route of infection with the route of vaccination is an attractive approach for the development of vaccines against TB.
- A number of successful mucosal vaccines against other diseases support the feasibility of this route for vaccination
- An increasing body of evidence in rodents, non-human primates, and humans highlight the immunogenicity and safety of mucosal TB vaccines.
- Better understanding of innate and adaptive immune responses against *M.tb* in the mucosa is required
- Future research should address the remaining key challenges, namely vaccine aerosolization, dosing and distribution within the respiratory mucosa
